# Macrophage reprogramming and functional plasticity in sepsis

**DOI:** 10.3389/fimmu.2026.1731805

**Published:** 2026-03-02

**Authors:** Tengyue Huang, Huiling Cheng, Qianru Zhao, Min Li

**Affiliations:** 1 Department of Critical Care Medicine, the Fourth Affiliated Hospital of School of Medicine, and International School of Medicine, International Institutes of Medicine, Zhejiang University, Yiwu, China; 2 Department of Echocardiography and Vascular Ultrasound Center, The First Affiliated Hospital, Zhejiang University School of Medicine, Hangzhou, Zhejiang, China

**Keywords:** epigenetics, immunity, macrophages, sepsis, therapy

## Abstract

Sepsis remains one of the leading causes of mortality worldwide, driven not by the infection itself but by a dysregulated host response that spirals into a cytokine storm and subsequent immune paralysis. This maladaptive immune reaction frequently culminates in life-threatening complications, including multiple organ failure and acute lung injury. Among the immune cells orchestrating this process, macrophages serve as pivotal sentinels of the innate immune system, coordinating inflammatory and reparative programs in response to microbial and endogenous cues. Increasing evidence now reveals that their behavior during sepsis is profoundly shaped by epigenetic regulation. Dynamic changes in DNA methylation, histone modifications, and non-coding RNAs fine-tune macrophage activation, polarization, and memory throughout the septic course. This review will dissect how these epigenetic programs dictate the initiation, progression, and resolution of sepsis, integrating recent discoveries to clarify underlying mechanisms and highlight promising epigenetic targets for therapeutic intervention.

## Introduction

Sepsis is a severe clinical syndrome primarily triggered by pathogenic infections. It arises from a dysregulated host response that progresses to systemic inflammatory response syndrome (SIRS) and multiple organ dysfunction syndrome (MODS) (1). Owing to its complex pathophysiology, rapid progression, and high mortality, sepsis remains one of the leading causes of death in intensive care units (ICUs) worldwide. Despite significant advances in understanding its pathogenesis and improving supportive care, effective and targeted therapeutic interventions are still lacking. Therefore, elucidating the underlying etiological and molecular mechanisms of sepsis is essential for developing precision medicine approaches and ultimately improving patient outcomes.

The innate immune system plays a pivotal role in the onset and progression of sepsis. Among innate immune cells, macrophages derived from the myeloid lineage serve as frontline defenders responsible for pathogen recognition, phagocytosis, and the initiation of inflammatory responses. With the rapid advancement of molecular biology and multi-omics technologies, the importance of epigenetic regulation in determining immune cell fate, maintaining functional plasticity, and mediating stress adaptation has become increasingly evident. Epigenetic regulation reshapes transcriptional programs without altering the DNA sequence, enabling macrophages to adapt their functions to changing microenvironmental cues. During sepsis, macrophages undergo extensive epigenetic reprogramming that modulates the inflammatory cascade, immune homeostasis, and tissue repair. In the later stages, however, aberrant epigenetic alterations may drive macrophage exhaustion and immune paralysis, predisposing patients to secondary infections, organ failure, and even death (2, 3).

A deeper understanding of how epigenetic programs shape macrophage function throughout the septic trajectory is pivotal for uncovering novel diagnostic and therapeutic opportunities. This review synthesizes emerging evidence on the central role of macrophages in sepsis, focusing on how epigenetic mechanisms coordinate their activation states, functional transitions, and immune regulation from early inflammation to immune resolution. By integrating insights from recent epigenomic and immunologic studies, we aim to offer a conceptual framework that links macrophage epigenetic remodeling to disease pathogenesis and potential avenues for precision therapy.

## 2 Pathophysiology of sepsis and the central role of macrophages

### 2.1 The pathological process of sepsis: from hyperinflammation to immunosuppression

The pathogenic mechanism of sepsis primarily originates from a dysregulated host response to pathogenic insults. In the early stage, pathogens and their components activate the innate immune system, triggering an excessive release of pro-inflammatory cytokines such as IL-1α, TNF-α, IL-1β, IL-6, and IFN-γ. This overwhelming inflammatory reaction, often referred to as a “cytokine storm,” leads to vascular dysfunction, increased endothelial permeability, and diffuse inflammatory injury affecting multiple organs (4). As the condition progresses, if this uncontrolled inflammation fails to resolve, the immune system gradually shifts from hyperactivation to exhaustion. During this transition, key immune cell including macrophages, monocytes, and lymphocytes experience profound functional impairment, giving rise to a state of “immune paralysis.” Consequently, patients become highly susceptible to secondary and opportunistic infections. Clinically, individuals with advanced sepsis often exhibit a pronounced immunosuppressive phenotype, characterized by recurrent or persistent infections that are difficult to control. This dynamic shift from an initial hyperinflammatory phase to a subsequent immunosuppressive or hyporesponsive phase represents a hallmark of sepsis pathogenesis and underlies its high mortality and complex clinical course (5).

### 2.2 Macrophage function and polarization in sepsis

Macrophages involved in sepsis arise from two major ontogenetic lineages: embryo-derived tissue-resident macrophages (TRMs) and bone marrow derived monocyte-derived macrophages (Mo-Macs). TRMs reside in barrier organs such as the lungs, liver, and spleen, where they self-renew and maintain tissue homeostasis through constant immune surveillance. In contrast, Mo-Macs are rapidly mobilized from the bone marrow in response to inflammatory stimuli and recruited to sites of infection, where they amplify local inflammation. These two macrophage populations display distinct functional characteristics, differentiation potential, and epigenetic landscapes. TRMs generally exhibit a more tolerant and regulatory phenotype, whereas Mo-Macs are predisposed to pro-inflammatory cytokine release and pathogen clearance (6). In the context of sepsis, impaired TRM function combined with excessive Mo-Mac activation creates a vicious cycle of inflammation and immune imbalance. Therefore, delineating macrophage ontogeny and targeting lineage-specific metabolic or epigenetic regulators may provide promising strategies for precision immunomodulatory therapy.

Macrophages are highly adaptable immune cells that perform diverse functions, including pathogen clearance, cytokine secretion, efferocytosis, and tissue remodeling. Their phenotype is remarkably plastic and can shift dynamically along an activation spectrum in response to environmental cues. Classically, macrophages are described as existing between two functional extremes: the pro-inflammatory M1 state and the anti-inflammatory or reparative M2 state, with undifferentiated M0 macrophages serving as intermediates. M1 macrophages dominate the early host response to infection, exhibiting strong antimicrobial and cytotoxic activities. They are characterized by the expression of inducible nitric oxide synthase (iNOS) and the production of pro-inflammatory mediators such as TNF-α, IL-1β, IL-6, and reactive oxygen species (ROS). In addition, M1 macrophages promote the formation of neutrophil extracellular traps (NETs), thereby amplifying inflammatory injury and tissue destruction. During the early phase of sepsis, excessive M1 activation triggers uncontrolled inflammation and cytokine overproduction, culminating in the so-called “cytokine storm” (7).

By contrast, M2 macrophages secrete anti-inflammatory mediators such as IL-10 and TGF-β, which suppress excessive immune activation while facilitating tissue repair and remodeling. This phenotype is essential for restoring immune homeostasis and promoting the resolution of inflammation (8). However, in the later stages of sepsis, a dominant shift toward M2 polarization contributes to profound immune paralysis and impaired pathogen clearance. Increasing evidence indicates that sepsis induces an “endotoxin tolerance” state, wherein monocytes and macrophages exhibit diminished cytokine production upon secondary stimulation, establishing a persistent immunosuppressive environment that perpetuates disease progression (9).

Overall, macrophages exist along a dynamic phenotypic continuum throughout the course of sepsis rather than fitting into discrete M1/M2 categories. While canonical signaling pathways including JAK-STAT, NF-κB, MAPK, cGAS-STING, and the inflammasome govern macrophage activation and polarization, growing evidence highlights that epigenetic mechanisms serve as equally pivotal, and possibly upstream, regulators of these functional transitions (10, 11).

## 3 Major types of macrophage epigenetic regulation and their roles in sepsis

### 3.1 DNA methylation

DNA methylation is primarily catalyzed by DNA methyltransferases (DNMTs) at cytosine-phosphate -guanine (CpG) dinucleotides. In general, hypermethylation of promoter or enhancer regions suppresses gene transcription, whereas hypomethylation facilitates gene activation. In the context of sepsis, DNA methylation plays a crucial role in shaping macrophage responses and disease progression.

Recent studies have demonstrated that *O6*-methylguanine-DNA methyltransferase (MGMT) expression in macrophages influences DNA methylation homeostasis and inflammatory output. Genetic deletion or pharmacologic inhibition of MGMT attenuates the progression of polymicrobial sepsis, suggesting that MGMT activity associates with the modulation of inflammatory severity (12). Compared with healthy individuals, monocytes from septic patients in a “tolerant” state exhibit globally increased DNA methylation, particularly within promoters of genes involved in monocyte activation (IL1A, CCL22, CCR2, STAT3), leading to transcriptional repression and functional hyporesponsiveness. These methylation patterns correlate with elevated plasma IL-6 and IL-10 levels and higher Sequential Organ Failure Assessment (SOFA) scores, linking DNA methylation to immune dysfunction and clinical severity (13). Mechanistically, inflammatory signaling pathways including Toll-like receptor and cytokine networks mediate these methylation process. In innate immune cells, LPS can reshape DNA methylation machinery occupancy at inflammatory loci; for example, in an endotoxin-tolerance model of human THP-1 monocytes, LPS-driven chromatin remodeling promotes recruitment of DNMT3A/3B to the *TNF* promoter, increasing CpG methylation and silencing transcription (14). Moreover, during the early phase of sepsis, key inflammatory genes (TNF, IL-6, IL1B) may undergo promoter hypomethylation in some models,enabling rapid and robust cytokine expression that drives hyperinflammation. As the disease progresses, these promoters may become re-methylated, leading to transcriptional silencing and reduced cellular responsiveness to secondary stimuli. This epigenetic reprogramming contributes to immune tolerance and sustained immunosuppression in late-stage sepsis (15, 16).

### 3.2 Histone modifications

Histone post-translational modifications represent a key layer of epigenetic regulation that fine-tunes gene transcription and plays a crucial role in the pathogenesis of sepsis. Among the numerous modification types acetylation, methylation, lactylation, phosphorylation, and ubiquitination histone acetylation and the recently identified lactylation have attracted particular attention for their functions in macrophage activation and inflammatory regulation (5, 17).

#### 3.2.1 Histone acetylation

Histone acetylation is a dynamic and reversible modification controlled by histone acetyltransferases (HATs) and histone deacetylases (HDACs) (18). This modification typically occurs on lysine residues within histone tails (e.g., H3K9, H4K16). Acetylation neutralizes the positive charge of histones, loosening their interaction with DNA and promoting an open chromatin configuration that facilitates transcription factor binding and gene activation (19).

In macrophages, histone acetylation closely interfaces with major inflammatory signaling pathways such as NF-κB. Conversely, HDACs remove acetyl groups, leading to chromatin condensation and transcriptional repression. Experimental models have shown that HDAC activity sustains hyperinflammation in sepsis. For instance, pharmacological inhibition of HDACs (including class I HDACs) in murine acute lung injury models markedly reduces inflammatory cytokine release and preserves tissue integrity, highlighting their pro-inflammatory role (20, 21).

Beyond chromatin regulation, non-histone acetylation also modulates macrophage activity. The deacetylase SIRT1, for example, deacetylates the Notch intracellular domain (NICD), suppressing downstream NF-κB signaling and improving survival in septic mice (22). Under inflammatory stress, the expression and enzymatic activity of HATs, HDACs, and sirtuins fluctuate dramatically, dynamically remodeling chromatin accessibility and shaping the transcriptional landscape of inflammation related genes.

#### 3.2.2 Histone lactylation

Lactate is a well-recognized biomarker of sepsis, and elevated circulating levels resulting from both overproduction and impaired clearance strongly correlate with disease severity and mortality. Beyond serving as a metabolic byproduct, lactate functions as a signaling molecule via receptors such as GPR81 and, more recently, as an epigenetic substrate that drives histone and non-histone protein lactylation (17). The discovery of histone lysine lactylation, identified through proteomic profiling and ^13^C-glucose tracing, established a novel paradigm linking cellular metabolism to chromatin regulation (23). Unlike acetylation, histone lactylation exhibits distinct kinetic patterns and cooperates with acetylation to modulate clock-controlled genes and maintain cellular homeostasis. Pathologically, lactylation of histone H3 (H3K18la) and the transcription factor Egr1 accelerates endothelial glycocalyx degradation in sepsis-induced acute lung injury (24). Elevated lactate also induces lactylation of the alarmin HMGB1 in macrophages, promoting its exosomal release, compromising endothelial integrity, and exacerbating systemic inflammation. In a feed-forward loop, hepatocyte-derived lactylated HMGB1 further activates macrophages and amplifies organ injury (25–27).

Collectively, the dynamic lactylation of histone and non-histone proteins serves as a crucial metabolic–epigenetic interface in sepsis. By integrating metabolic cues into transcriptional regulation, this modification orchestrates the balance between inflammatory activation and resolution, and represents a promising target for therapeutic intervention.

### 3.3 Non-coding RNA regulation

Beyond DNA methylation and histone modifications, epigenetic control in sepsis extends to non-coding RNAs (ncRNAs), including microRNAs (miRNAs), long non-coding RNAs (lncRNAs), and circular RNAs (circRNAs). These ncRNAs fine-tune gene expression by interacting with chromatin modifiers, transcription factors, and signaling pathways.

Several miRNAs such as miR-155, miR-146a, and miR-30 critically regulate macrophage polarization between pro-inflammatory (M1) and anti-inflammatory (M2) states, thereby shaping immune responses during sepsis (28, 29). In sepsis-associated acute kidney injury (SA-AKI), extracellular vesicles derived from endothelial progenitor cells (EPC-EVs) deliver miR-93-5p, which exerts protective effects through the KDM6B/H3K27me3/TNF-α axis (30). Within the macrophage epigenetic network, lncRNAs and circRNAs act as regulatory hubs that orchestrate immune responses via chromatin remodeling, transcriptional interference, and post-transcriptional modulation. The lncRNA MALAT1 exemplifies this role: its expression is markedly upregulated in patients with late-onset sepsis, in PBMCs from septic mice, and in LPS stimulated THP-1 macrophages, correlating strongly with disease severity (31). Similarly, circRNAs such as circC3P1 modulate inflammation by sponging specific miRNAs (e.g., miR-21). In sepsis-induced acute lung injury, circC3P1 attenuates cytokine production and apoptosis through miR-21 regulation (32).

Emerging evidence also suggests extensive cross-talk between ncRNAs and classical epigenetic modifications. miRNAs can target histone-modifying enzymes, while lncRNAs and circRNAs can influence DNA methyltransferase recruitment or histone methylation patterns. Together, DNA methylation, histone modifications, and ncRNA networks form an integrated epigenetic circuitry that enables macrophages to rapidly adapt their transcriptional programs to the evolving inflammatory milieu of sepsis.

## 4 Epigenetic regulation of core macrophage biological processes

### 4.1 Metabolic–epigenetic coupling in macrophage reprogramming during sepsis

During the acute systemic infection of sepsis, macrophages central effectors of the innate immune system undergo profound metabolic and functional reprogramming. Recent studies highlight that several tricarboxylic acid (TCA) cycle intermediates serve as key epigenetic modulators, directly linking cellular metabolism to transcriptional control in macrophages. Upon early infection, LPS-TLR4 signaling rapidly activates M1 macrophages, enhancing glycolytic flux and remodeling the TCA cycle. This metabolic rewiring leads to the accumulation of intermediates such as succinate and citrate. Succinate inhibits prolyl hydroxylases (PHDs), thereby stabilizing HIF-1α and driving the expression of pro-inflammatory cytokines such as IL-1β. It also reinforces inflammatory transcriptional programs through histone succinylation (33). Meanwhile, citrate-derived acetyl-CoA provides the substrate for histone acetylation, further amplifying the expression of inflammatory genes.

Sustained inflammation imposes metabolic stress characterized by mitochondrial ROS accumulation and fluctuating metabolite levels, ultimately driving the transition of macrophages from a glycolytic M1 phenotype to an oxidative M2 phenotype. M2 macrophages display enhanced mitochondrial respiration and upregulate anti-inflammatory and reparative genes such as Arg1 and PPARγ, a process tightly regulated by the mTOR AMPK axis and other metabolic sensors (34). Metabolites also act as cofactors or inhibitors of chromatin modifying enzymes. Succinate competitively inhibits α-ketoglutarate (α-KG) dependent demethylases such as KDM5 and JMJD3, leading to the accumulation of activating histone marks (e.g., H3K4me3) and maintenance of a pro-inflammatory state (35). In contrast, α-KG promotes demethylase activity, erasing repressive marks such as H3K27me3 to facilitate transcription of M2-associated genes including IL10. Moreover, lactate the terminal product of glycolysis has recently been identified as a potent epigenetic signal. It induces histone lactylation, which activates reparative gene expression and supports the development of “trained immunity” during the resolution phase of inflammation (36).

Collectively, these intertwined metabolic and epigenetic processes define the “metabolism epigenetics function” axis in macrophages during sepsis. Accordingly, targeting key metabolic enzymes, such as pyruvate kinase M2 (PKM2), a rate-limiting glycolytic enzyme toggle controlling pyruvate production and inflammatory polarization, or isocitrate dehydrogenase (IDH), a tricarboxylic acid (TCA) cycle enzyme that regulates α-ketoglutarate (α-KG) availability and thus influences α-KG dependent dioxygenases involved in histone and DNA demethylation, has emerged as a promising strategy to restore immune balance. In parallel, pharmacologically manipulating epigenetic regulators (e.g., BET proteins and HDACs) may provide complementary approaches to reshape macrophage transcriptional programs and improve outcomes in sepsis (2, 37).

### 4.2 Crosstalk between autophagy and epigenetic regulation in sepsis

Autophagy, a fundamental cellular process for maintaining homeostasis under stress, emerges as a critical regulator of immune inflammation during sepsis. In response to oxidative stress, microbial invasion, and metabolic perturbation, autophagy acts as a protective mechanism that limits uncontrolled inflammation and prevents cellular damage by coordinating organelle quality control, pathogen clearance, and inflammatory signaling.

Recent studies have uncovered a tight interconnection between autophagy and epigenetic regulation. Histone modifications (e.g., H3K4me3, H3K27ac) and DNA methylation patterns directly influence the transcription of autophagy related genes such as ATG5 *and* MAP1LC3B, thereby affecting macrophage polarization and the magnitude of the inflammatory response (38, 39). In parallel, metabolic intermediates including α-ketoglutarate, succinate, and fumarate regulate the activity of histone demethylases (e.g., KDM, KDM6B) and DNA-modifying enzymes (e.g., TET, DNMT), establishing a “metabolism epigenetics–autophagy” axis that shapes immune memory and inflammatory outcomes (40–44). Conversely, autophagy also feeds back to modulate epigenetic processes. By regulating intracellular metabolite levels such as NAD^+^, S-adenosylmethionine (SAM), and acetyl-CoA, autophagy indirectly influences histone acetylation and methylation states, forming intricate metabolic epigenetic feedback loops (45–47).

Taken together, autophagy not only safeguards immune equilibrium during sepsis but also acts as an essential integrator of metabolic and epigenetic cues that determine immune cell fate and function. Elucidating this “autophagy epigenetics” network will provide novel insights into the mechanisms of immune regulation and may uncover promising therapeutic targets for restoring homeostasis in sepsis.

### 4.3 Epigenetic regulation of macrophage migration and residence

Macrophages are highly dynamic immune cells that couple migratory responsiveness with tissue residency to maintain immune surveillance and homeostasis. During systemic infections such as sepsis, they sense chemotactic gradients, mobilize from the circulation or bone marrow, and settle within specialized tissue niches where they integrate microenvironmental signals to eliminate pathogens, modulate inflammation, and promote repair. The efficiency of these processes is governed not only by chemokine and adhesion pathways but also by epigenetic programs that shape macrophage phenotype and functional specialization.

The recruitment of macrophages is primarily governed by chemokine–receptor interactions such as CCL2-CCR2, CX3CL1-CX3CR1, and CXCL12-CXCR4 (48). The chromatin configuration of these receptor genes is highly dynamic and directly influences migratory potential. In the early stages of inflammation, genes such as CCR2 are transcriptionally activated through the cooperation of histone modifications and transcription factors. The enrichment of activating histone marks (e.g., H3K4me3) in promoter regions facilitates NF-κB binding, while the demethylase JMJD3 removes repressive marks (e.g., H3K27me3), together promoting efficient monocyte and macrophage recruitment to infected tissues (49–51). By contrast, during the late or immunosuppressive phase of sepsis, inhibitory histone marks such as H3K9me3 and H3K27me3 accumulate at the promoter regions of chemokine receptor genes, leading to CCR2 downregulation. As a result, monocytes lose responsiveness to chemotactic signals and fail to reach sites of infection, which facilitates pathogen dissemination and aggravates organ dysfunction (51, 52).

Following recruitment, macrophage extravasation and stable tissue retention depend on adhesion molecules (e.g., ICAM1, VCAM1) and integrins (e.g., CD11b/CD18) (53, 54). Epigenetic modulation also governs the expression of these factors. For instance, histone deacetylase inhibitors have been shown to alter endothelial adhesion programs (e.g., VCAM-1/ICAM-1), thereby modulating monocyte/macrophage adhesion and recruitment during inflammation (55). In contrast, during the immunosuppressive stage of sepsis, epigenetic reprogramming characterized by H3K27me3 enrichment can suppress the expression of key adhesion and integrin genes. This disruption of the “migration–residence” balance increases macrophage motility but compromises their anchoring capacity, preventing the formation of stable functional clusters at infection sites. Consequently, local immune surveillance and tissue repair are impaired, predisposing to secondary infections and organ damage (56).

Macrophage migration and residency are further tuned by cues from the local microenvironment. In the acute inflammatory phase, hypoxia and pro-inflammatory mediators activate HIF-1α, which can promote chemokine and chemokine receptor programs (e.g., CXCR4) through transcriptional regulation, thereby facilitating macrophage recruitment to inflammatory foci (9, 57, 58). As inflammation transitions into the resolution and repair stage, the histone demethylase JMJD3 is induced. By removing the repressive histone mark H3K27me3, JMJD3 activates repair-associated gene programs such as CD163 *and* Irf4, promoting macrophage differentiation toward an M2-like or tissue-resident phenotype that supports tissue regeneration and strengthens anchorage within the tissue microenvironment (59, 60).

**Figure 1:**
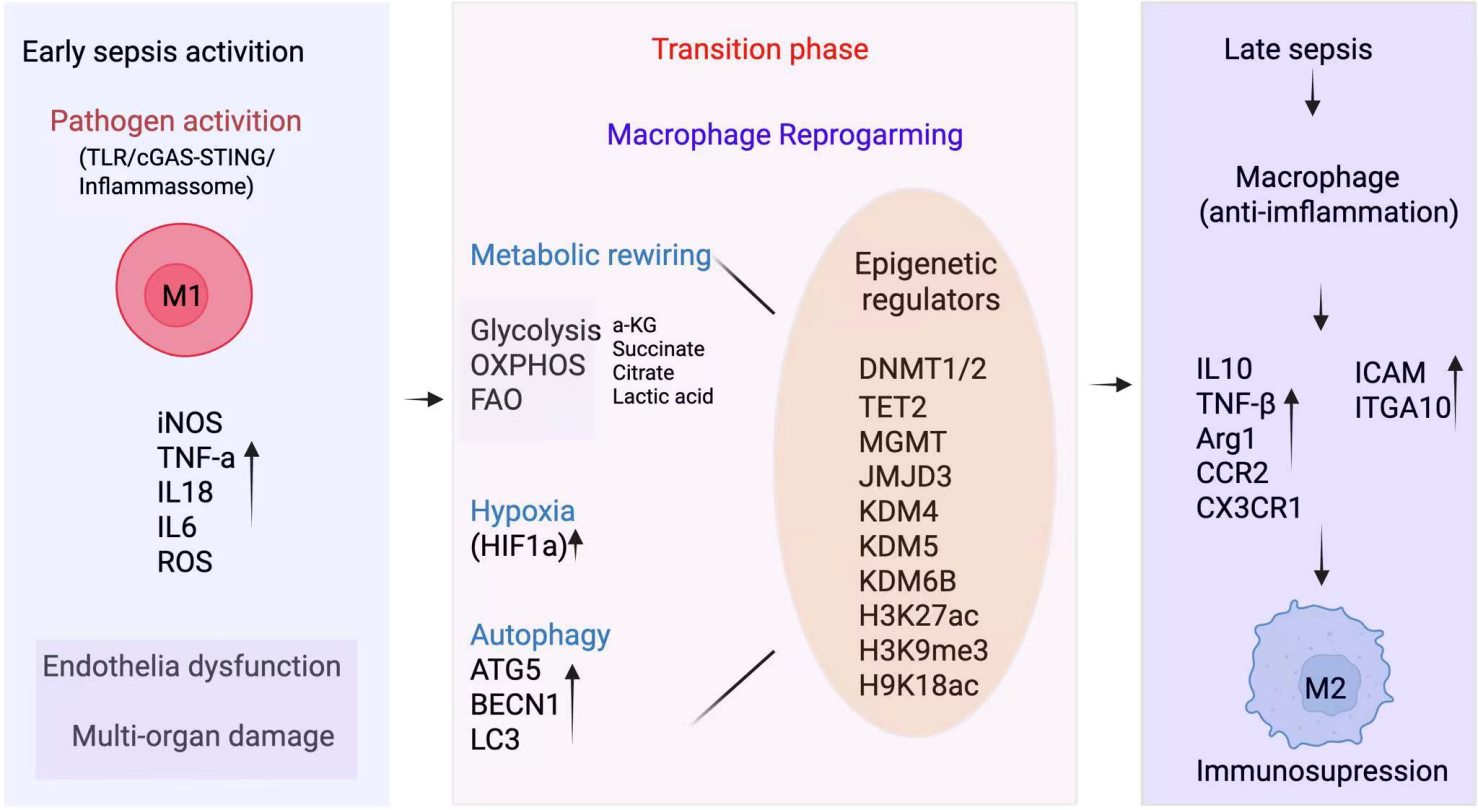
Dynamic macrophage reprogramming in sepsis: from hyperinflammation to immunosuppression.

Taken together, the epigenetic status of macrophages plays a central role in orchestrating their recruitment, migration, and tissue residency during sepsis. Through dynamic chromatin remodeling, macrophages integrate systemic chemotactic signals with local environmental cues, maintaining a delicate balance between motility and retention. This balance is fundamental for effective pathogen clearance, immune regulation, and tissue restoration throughout the complex immunopathological course of sepsis. These findings highlight that epigenetic alterations in macrophages profoundly affect both their functional states and the progression of sepsis (**Figure 1**). Thus, in-depth exploration of the underlying epigenetic mechanisms and the development of targeted epigenetic interventions holds considerable promise for improving sepsis outcomes.

Sepsis progression is characterized by a transition from an early hyperinflammatory phase to a later immunosuppressive state, accompanied by profound macrophage functional plasticity. In the early activation stage, pathogen-derived signals and danger-associated molecular patterns activate macrophages through innate immune pathways including TLR signaling, cGAS-STING, and inflammasome activation, promoting an M1-like phenotype with increased iNOS expression and elevated production of pro-inflammatory cytokines (TNF-α, IL-1β, IL-6) and reactive oxygen species (ROS). This exaggerated inflammatory response contributes to endothelial dysfunction, tissue injury, and multi-organ damage. During the transition phase, macrophages undergo metabolic rewiring, shifting glycolysis, oxidative phosphorylation (OXPHOS), and fatty acid oxidation (FAO), accompanied by accumulation of metabolic intermediates such as succinate, citrate, lactate, and α-ketoglutarate (α-KG). These metabolites serve as signaling molecules that directly influence epigenetic remodeling. Concurrently, multiple epigenetic regulators including DNA methylation modifiers (DNMT1/2, TET2, MGMT), histone demethylases (JMJD3, KDM4, KDM5, KDM6B), and histone modification states (H3K27ac, H3K9me3, H3K18ac) reshape chromatin accessibility and transcriptional programs. Autophagy-related pathways (ATG5, BECN1, LC3) also integrate metabolic stress responses and epigenetic regulation to modulate macrophage survival and inflammatory output. In the late stage of sepsis, macrophages adopt an M2-like anti-inflammatory phenotype characterized by increased expression of immunoregulatory mediators such as IL-10 and Arg1, altered chemokine receptor profiles (CCR2, CX3CR1), and adhesion-related molecules (ICAM, ITGA10), ultimately contributing to immune paralysis and increased vulnerability to secondary infections. Together, this schematic highlights the coordinated metabolic–epigenetic–autophagy axis that drives macrophage state transitions across sepsis stages and provides a conceptual framework for therapeutic targeting.

## 5 Clinical translation and therapeutic targeting

### 5.1 Diagnostic and prognostic epigenetic biomarkers

Accumulating evidence directly links the epigenetic remodeling of circulating monocytes and macrophages to sepsis outcomes. In patients, aberrant DNA methylation correlates with inflammatory cytokine release and organ failure, suggesting that the monocyte methylome can serve as a molecular proxy for immune dysregulation. Epigenome-wide association studies have further identified methylation changes that correspond to specific clinical scores, underscoring their potential for patient stratification (61). This epigenetic shift drives a ‘tolerant’ or ‘immunoparalyzed’ phenotype by transcriptionally silencing key immune activation pathways. This functional impairment is now understood to be a critical factor in late-stage sepsis mortality and the heightened risk of secondary infections.

Beyond intracellular methylomic alterations, circulating nucleic acids present a compelling platform for clinical diagnostics. Elevated levels of plasma cell-free DNA (cfDNA) and mitochondrial cfDNA (mt-cfDNA) correlate quantitatively with tissue injury, organ dysfunction scores, and mortality risk, establishing them as robust, minimally invasive prognostic indicators (62, 63). Crucially, the utility of cfDNA extends beyond simple quantification. Advanced techniques, such as bisulfite-based methylation sequencing or targeted methylome assays, enable the deconvoluting of tissue-of-origin signals and the detection of immune-related epigenetic shifts. These insights provide a mechanistic window into systemic injury patterns and inflammatory states (64). Such epigenetic profiling may significantly complement conventional biomarkers by capturing the dynamic molecular landscape of the septic trajectory.

From a translational standpoint, several assay formats are under investigation to implement epigenetic biomarkers in clinical settings. These include genome scale or targeted DNA methylation profiling of blood leukocytes, assays based on chromatin accessibility or modifications, and the measurement of circulating non-coding RNAs in serum or plasma. Specific microRNAs, such as miR-155, miR-146a, miR-223, and miR-21, are consistently implicated in regulating innate immune activation and endotoxin tolerance. This provides a strong rationale for developing serum/plasma miRNA panels, analyzed via qPCR- or ddPCR-based platforms, to serve as complementary biomarkers for defining a patient’s immune status (65). Conceptually, integrating DNA methylation or miRNA signatures with established clinical endotyping frameworks could prove instrumental in distinguishing between hyperinflammatory and immunosuppressive disease trajectories. This differentiation is crucial for guiding targeted immunomodulatory therapies (66).

### 5.2 Targeting epigenetic enzymes and readers

Preclinical evidence increasingly supports targeting epigenetic regulators to recalibrate dysregulated immune responses in sepsis. Among epigenetic “writers,” DNMT inhibition has demonstrated efficacy in polymicrobial models. In cecal ligation and puncture (CLP) assays, early administration of decitabine (0.5 mg/kg s.c.) curtailed systemic cytokine release and bolstered survival by suppressing hyperactive NF-κB signaling (60, 67). However, clinical translation remains hampered by systemic toxicities; decitabine is associated with dose-limiting myelosuppression, and DNMT-trapping agents pose genotoxic risks (68). These constraints necessitate the development of abbreviated dosing regimens or immune-cell-targeted delivery systems.

Beyond DNA methylation, HDAC inhibition offers an alternative therapeutic axis. While Trichostatin A (TSA) effectively reduced inflammatory damage in murine lung injury models (69, 70), the risk of hematologic and cardiac adverse effects from broad HDAC blockade limits its systemic viability (5). Targeting epigenetic ‘readers’ provides a potent strategy to decouple histone acetylation from pro-inflammatory transcription. Specifically, BET proteins (e.g., BRD4) serve as molecular scaffolds that recruit the transcriptional machinery to activated chromatin. While the BET inhibitor JQ1 (50 mg/kg i.p.) effectively quells macrophage-driven cytokine storms and improves survival in lethal endotoxemia (71, 72), its clinical utility is constrained by narrow therapeutic windows and systemic cardiovascular/metabolic toxicities (73). Finally, noncanonical regulators may provide additional entry points: myeloid MGMT deletion or inhibition induced TNF-α promoter hypermethylation and alleviated polymicrobial sepsis severity (74). Collectively, these findings highlight the promise of epigenetic therapy while underscoring the importance of dosing windows, cell specificity, and toxicity control for clinical translation.

### 5.3 Non-coding RNA-based therapeutics

Therapeutic modulation of non-coding RNAs (ncRNAs) offers a programmable framework to recalibrate macrophage polarization and restore immune homeostasis. Preclinical studies demonstrate that miRNA mimics or antagomirs effectively blunt cytokine-mediated tissue injury (65). Specifically, antisense inhibition of miR-155, a master regulator of M1 polarization can curtail hyper-inflammation (75), while the delivery of miR-146a serves to dampen excessive NF-κB signaling (65). The translational viability of these RNA therapeutics is significantly bolstered by lipid nanoparticles (LNPs) and engineered extracellular vesicles (EVs), which resolve inherent stability issues and enhance macrophage-specific uptake (76). For instance, EPC-derived EVs loaded with miR-93-5p have been shown to mitigate sepsis-induced acute kidney injury by remodeling the KDM6B/H3K27me3/TNF-α epigenetic axis, providing a template for vesicle-mediated precision intervention (30). And circular RNAs (circRNAs) also function as critical regulatory hubs. Targeting the lncRNA MALAT1 may curb NF-κB mediated macrophage activation (31), while circRNAs like circC3P1 protect against acute lung injury by sequestering miR-21 (32). While ncRNAs offer a versatile therapeutic platform, optimizing delivery kinetics and safety profiles remains a prerequisite for clinical adoption.

### 5.4 Immunometabolic and autophagic interventions

The intrinsic coupling between cellular metabolism and chromatin remodeling provides a tractable entry point to indirectly redirect macrophage programs (77). This regulatory link is primarily attributable to shifts in metabolic flux, such as the transition toward aerobic glycolysis or altered fatty acid oxidation which fundamentally change the intracellular availability of acetyl-CoA and NAD^+^. Since these metabolites function as obligatory substrates and cofactors for histone acetyltransferases (HATs) and sirtuins (HDACs), their systemic fluctuations directly dictate the epigenetic landscape and subsequent macrophage polarization (78, 79).

Furthermore, the therapeutic potential of this axis is underscored by the emergence of lactate as a key signaling driver rather than a mere metabolic byproduct. Due to its ability to promote histone lactylation (e.g., H3K18la), lactate acts as a direct mechanistic bridge between metabolic rewiring and the transcriptional reprogramming of inflammatory genes (80, 81). Following this causal chain, pharmacological interventions that restrict lactate production including glycolysis inhibitors (2-DG), LDH inhibitors (oxamate), and PDK inhibitors (dichloroacetate) can effectively prevent pathological lactylation and restrain hyper-inflammatory amplification (79, 80). Beyond direct inhibition, restoring metabolic-epigenetic equilibrium via AMPK activators (e.g., metformin) or monocarboxylate transporter (MCT) inhibitors (e.g., AZD3965) has shown promise in recalibrating immune homeostasis (77, 78). By virtue of balancing these metabolic sensors, such interventions ensure that the epigenetic machinery receives the appropriate metabolic cues to promote the resolution of inflammation rather than its persistence.

Pharmacologic regulation of autophagy serves as a complementary approach to manage macrophage-driven injury (82). Agents such as Rapamycin, 3-MA, and various bioactive natural products (e.g., Artemisia annua extracts, fisetin, emodin) modulate ROS levels and inflammasome assembly. By preventing excessive M1 polarization or preserving protective macrophage functions, these compounds improve survival outcomes in experimental sepsis (8, 83). Detailed comparisons of these epigenetic targets are provided in **Table 1**.

**Table 1:** Therapeutic potential of key epigenetic mechanisms targeting macrophages in sepsis.

Epigenetic mechanism	Specific target	Representative drugs interventions	Main effects function in sepsis	Research stage
DNA Methylation	DNMT	Decitabine	Alleviates cytokine storm and improves survival in septic mice	Preclinical
	MGMT	MGMT inhibitors	Induces hypermethylation of TNF-α promoter, suppresses its overexpression, and alleviates sepsis severity	Preclinical
Histone Acetylation	HDAC	Trichostatin A	Reduces inflammation and improves tissue damage (e.g., in acute lung injury)	Preclinical
	BET Proteins	JQ1	Inhibits production of key pro-inflammatory cytokines (IL-6, TNF-α); significantly improves survival in lethal endotoxemia models	Preclinical
Non-coding RNA	miR-155	Inhibitors	Suppresses pro-inflammatory macrophage polarization and attenuates hyperinflammatory response	Preclinical
	miR-146a	Mimics	Enhances anti-inflammatory response and improves outcomes	Preclinical
Lactylation	Histone Lactylation	Lactate clearance agents, LDH inhibitors	Modulates inflammatory resolution programs and macrophage phenotypes via histone lactylation	Concept Validation

### 5.5 Therapeutic windows and precision stratification

The efficacy of epigenetic interventions in sepsis is strictly governed by the therapeutic time window and the heterogeneity of patient-specific immune states. Due to the dynamic evolution of sepsis, which typically transitions from an acute hyper-inflammatory “cytokine storm” to a protracted state of immunoparalysis, the macrophage transcriptional and epigenetic landscapes undergo continuous remodeling (5). Consequently, a “one-size-fits-all” therapeutic strategy is fundamentally flawed (84). Attributable to these divergent immune phases, epigenetic suppressors such as HDAC or BET inhibitors are most rational during the early inflammatory surge. Conversely, as the pathology progresses into the immunosuppressive phase, the therapeutic objective must pivot toward restoring immune competence through interventions that reverse epigenetic silencing, such as DNMT inhibition or metabolic reprogramming (85, 86).

Realizing the potential of precision epigenetic therapy is therefore contingent upon the establishment of robust stratification frameworks that integrate dynamic molecular biomarkers with longitudinal clinical data (87). By virtue of high-throughput technologies, potential real-time tools now include PBMC DNA methylation profiles, mass spectrometry-quantified circulating H3K18 lactylation, and the miR-155/miR-146a ratio (88–90). Mapping these molecular readouts alongside conventional clinical indices, such as SOFA scores and lactate clearance, enables the construction of data-driven “immune endotypes” (91, 92). Such a framework ensures that epigenetic interventions are not merely applied broadly, but are precisely matched to the patient’s specific phase of immune dysregulation, a critical prerequisite for the successful clinical translation of precision medicine in sepsis.

## 6 Discussion and future perspectives

Sepsis is a multifaceted syndrome driven by the interplay between pathogenic infection and dysregulated host immunity, characterized by a transition from an early hyperinflammatory phase to a subsequent immunosuppressive phase. Macrophages lie at the center of this continuum, acting both as amplifiers of acute inflammation and as mediators of immune paralysis. Increasing evidence indicates that epigenetic mechanisms govern these functional transitions, determining whether macrophages drive destructive inflammation or facilitate immune resolution (93).

While targeting macrophage epigenetic programs offers a compelling therapeutic avenue for sepsis, its clinical translation remains hampered by several formidable barriers. A primary concern is the broad genomic activity of current epigenetic agents, such as HDAC and BET inhibitors. These drugs often trigger unintended transcriptional rewiring outside of inflammatory pathways, risking systemic toxicity or the collapse of immune homeostasis (69–72). Overcoming this requires a shift toward tissue- or cell-type-restricted delivery, where platforms like macrophage-targeted nanoparticles could provide the necessary precision to minimize off-target effects. The clinical landscape is further complicated by the stark inter-patient heterogeneity of epigenetic profiles. This diversity suggests that a “one-size-fits-all” approach is unlikely to succeed; instead, we must integrate multi-omics datasets by encompassing epigenomic, transcriptomic, and metabolomic layers and build predictive frameworks capable of identifying patient subsets most responsive to intervention (61, 66, 89). Real-time clinical management could be further refined through the longitudinal monitoring of circulating biomarkers, such as H3K18 lactylation and miR-146a expression, allowing for dynamic, biomarker-driven immunomodulation. Ultimately, the future of precision sepsis therapy hinges on our ability to use big data analytics and systems-level integration to map the complex immune states of macrophage populations.

In conclusion, macrophages in sepsis function as both initiators and regulators of immune dynamics, orchestrating the delicate balance between inflammation and tolerance. Their epigenetic regulation not only underpins this functional plasticity but also provides a promising framework for therapeutic innovation. With continuous advancements in high-throughput sequencing, single-cell profiling, and epigenetic editing technologies, interventions targeting macrophage epigenetics are poised to become a central pillar of future sepsis therapy offering new avenues for precision treatment and improved patient outcomes. Most of the current mechanistic insights are derived from murine models of sepsis, and the translational relevance of these epigenetic pathways in human patients requires further validation. The pleiotropic effects of epigenetic drugs pose a significant challenge for their clinical application in sepsis, necessitating the development of more targeted delivery systems.

## Glossary

SIRS: Systemic Inflammatory Response Syndrome
MODS: Multiple Organ Dysfunction Syndrome
ICU: Intensive Care Unit
TRMs: Tissue Resident Macrophages
Mo-Macs: Monocyte Derived Macrophages
M1: Classically activated macrophages
iNOS: Inducible Nitric Oxide Synthase
ROS: Reactive Oxygen Species
NETs: Neutrophil Extracellular Traps
M2: Alternatively activated macrophages
DNMTs: DNA Methyltransferases
CpG: Cytosine-phosphate-Guanine
MGMT: O6-methylguanine-DNA methyltransferase
LPS: Lipopolysaccharide
SOFA: Sequential Organ Failure Assessment
HATs: Histone Acetyltransferases
HDACs: Histone Deacetylases
BET: Bromodomain and Extra Terminal
SA-AKI: Sepsis-Associated Acute Kidney Injury
EPC-EVs: Endothelial Progenitor Cell derived Extracellular Vesicles
TCA: Tricarboxylic Acid Cycle
α-KG: Alpha-Ketoglutarate;PHDs, prolyl hydroxylases
HIF-1α: Hypoxia inducible factor 1-α
TET: Ten eleven translocation
BRD4: Bromodomain-containing protein 4
SAM: S-adenosylmethionine
NAD: Nicotinamide adenine dinucleotide
ICAM-1: Intercellular adhesion molecule-1
VCAM-1: Vascular cell adhesion molecule-1
CCL2: C-C motif chemokine ligand 2
CCR2: C-C chemokine receptor type 2
CX3CL1: C-X3-C motif chemokine ligand 1
CX3CR1: C-X3-C motif chemokine receptor 1
CXCL12: C-X-C motif chemokine ligand 12
CXCR4: C-X-C chemokine receptor type 4
3-MA: 3-Methyladenine
PKM2: Pyruvate kinase M2**;** IDH, Isocitrate dehydrogenase**;** LDH, Lactate dehydrogenase**;** PDK, Pyruvate dehydrogenase kinase**;** AMPK, AMP-activated protein kinase**;** mTOR, mechanistic target of rapamycin**;** MCT, Monocarboxylate transporter.
